# Complete Coding Sequences of Five Dengue Virus Type 2 Clinical Isolates from Venezuela Obtained through Shotgun Metagenomics

**DOI:** 10.1128/genomeA.00545-18

**Published:** 2018-06-21

**Authors:** Erley Lizarazo, Natacha Couto, Maria Vincenti-Gonzalez, Erwin C. Raangs, Thomas Jaenisch, Alex W. Friedrich, Adriana Tami, John W. Rossen

**Affiliations:** a Department of Medical Microbiology, University Medical Center Groningen, University of Groningen, Groningen, The Netherlands; b University of Heidelberg, Heidelberg, Germany; c Departamento de Parasitología, Facultad de Ciencias de la Salud, Universidad de Carabobo, Valencia, Venezuela

## Abstract

Dengue is a disease endemic in Latin American countries, like Venezuela, and has become one of the most important public health problems. We report five complete coding sequences of dengue virus serotype 2 (DENV-2) isolated from DENV-infected patients in Venezuela. Phylogenetic analysis placed the isolates within the American/Asian genotype.

## GENOME ANNOUNCEMENT

Dengue virus (DENV) infection continues to be one of the most prevalent arboviral diseases in tropical and subtropical regions, with an estimated burden of 390 million cases/year worldwide (1). Infection by any of the four DENV serotypes (DENV-1 to DENV-4) can lead to a wide spectrum of clinical outcomes, ranging from asymptomatic cases, mild disease as a flu-like syndrome (dengue without warning signs), to a more severe form of disease (dengue with warning signs or severe dengue) (1–3). Severe disease is frequently associated with the Asian DENV-2 and DENV-3 genotypes with secondary infections (4). To date, there is no fully successful vaccine or specific treatment for DENV infection (5, 6).

We report five complete coding sequences of dengue virus serotype 2 (DENV-2) isolated from DENV-infected patients in Venezuela in 2015, of whom two patients had dengue with warning signs. The study was approved by the ethics review committee of the Biomedical Research Institute, Carabobo University [Aval Bioetico numbers CBIIB(UC)-014 and CBIIB-(UC)-2013-1], Maracay, Venezuela, and the Ethics, Bioethics and Biodiversity Committee of the National Foundation for Science, Technology and Innovation, Caracas, Venezuela. For sequencing, RNA isolation was performed with the QIAamp viral RNA isolation kit (Qiagen, Hilden, Germany). Libraries were prepared with the TruSeq V2 RNA (Illumina, San Diego, CA, USA), which includes a cDNA synthesis step. Sequencing was performed on a MiSeq instrument with the MiSeq reagent kit version 2 (Illumina) that generated 150-bp paired-end reads. The sequences were assembled and analyzed using the CLC Genomics Workbench version 10.1.1 software (Qiagen). Genome annotation was performed using the plugin MetaGeneMark version 1.4.

The complete open reading frame (ORF) of the DENV-2 polyprotein was obtained in all samples with a coverage of greater than 177-fold. The lengths of the genomes sequenced were 10,694 nucleotides (nt), 10,619 nt, 10,712 nt, 10,711 nt, and 10,704 nt, with the lengths of the 5ʹ/3ʹ untranslated regions being 87/431 nt, 87/356 nt, 84/452 nt, 86/449 nt, and 77/451 nt, respectively. These are slightly shorter than the 96/451 nt reported for the DENV-2 reference genome (GenBank accession number NC_001474). The phylogenetic analysis based on the complete ORF using the maximum likelihood method revealed that the isolates belong to the American/Asian genotype. Strains clustered in two different subpopulations, sharing a common ancestor within the Venezuelan clade.

Molecular surveillance to monitor circulating DENV-2 strains in Venezuela is lacking nowadays, and any information about current or past circulating strains has not been reported since 2008 (7). The replacement of less virulent strains by the (re)emergence of more virulent strains or *in situ* recombination has happened before in Venezuela; consequently, such potential changes should be monitored to reveal evolutionary trends (8). The sequences of DENV-2 described in this work will help follow up on the molecular epidemiology of DENV in Venezuela. In addition, these genome sequences add to the knowledge of the current DENV-2 diversity and endemicity, which are important for developing future accurate vaccines.

### Accession number(s).

The complete coding sequences of the five DENV-2 strains described here have been deposited in GenBank under the accession numbers MH069495 (DENV-2/VE/IDAMS/921099), MH069496 (DENV-2/VE/IDAMS/921096), MH069497 (DENV-2/VE/IDAMS/921095), MH069498 (DENV-2/VE/IDAMS/910121), and MH069499 (DENV-2/VE/IDAMS/910105).
